# Inflammatory Response and Electrocardiographic Examination in Horses Vaccinated against Equine Herpesvirus (Ehv-1)

**DOI:** 10.3390/ani12060778

**Published:** 2022-03-19

**Authors:** Vito Biondi, Alessandra Landi, Michela Pugliese, Giordana Merola, Annamaria Passantino

**Affiliations:** 1 Department of Veterinary Sciences, University of Messina, 98168 Messina, Italy; vito.biondi@unime.it (V.B.); giordana.merola@gmail.com (G.M.); passanna@unime.it (A.P.); 2 Veterinary Practitioner, 98168 Messina, Italy; alelandi1612@gmail.com

**Keywords:** equine herpesvirus, cardiac troponin I, C-reactive protein, serum amyloid A, arrhythmias

## Abstract

**Simple Summary:**

Equine herpesvirus 1 (EHV-1) is an alphaherpesvirus that infects horses, causing respiratory, neurologic, and abortion syndromes in pregnant mares. Vaccination induces an immune response that reduces the risk of infection, the severity of clinical signs, and viral excretion. This study aimed to evaluate and describe the clinical and electrocardiographic findings, and changes in cardiac troponin I (cTnI) and inflammatory biomarkers (serum amyloid A (SAA) and C-reactive protein (CRP)) occurring after vaccination against herpesvirus in healthy horses.

**Abstract:**

This study aimed to evaluate possible abnormalities in electrocardiographic findings, and changes in cardiac troponin I (cTnI) and inflammatory biomarkers (serum amyloid A (SAA) and C-reactive protein (CRP)) after inactivated herpesvirus vaccine administration. Eighteen healthy horses were included. All animals were vaccinated with Pneumoequine^®^ (Merial, France) according to the protocol provided by the manufacturer. They were evaluated 1 day before the first dose of vaccination (D0), and 7 days (D1) and 14 days (D2) afterwards. At D0, D1, and D2, a blood sample was taken for the evaluation of SAA, cTnI, and CRP. An electrocardiographic examination was also performed. The data obtained suggested the possible involvement of the myocardium following vaccination against herpesvirus 1, mostly related to an inflammatory response.

## 1. Introduction

Equine herpesvirus-1 (EHV-1) is a highly contagious virus belonging to the family Herpesviridae with worldwide distribution. It is a cause of serious economic losses through the occurrence of abortion in pregnant mares, neonatal death in foals, respiratory disease in young performance horses, and myeloencephalopathy [1,2,3,4].

EHV-1 is usually transmitted through the inhalation of infected aerosols or direct contact with infected secretions (saliva, nasal discharge) [2]. Fetal or placental tissues that contain high concentrations of the virus are considered a potential source of infection [5,6,7,8]. The infection typically results in latent infection during the first weeks or months of life [7]. Stress factors such as intense physical activity, transport, suppressed immunity, and fatigue induce subsequent viral reactivation and shedding with the occurrence of clinical signs [4].

Vaccination is considered the main means of preventing the occurrence of abortion or neurologic disease, stimulating the immune response and consequently reducing or eliminating cell-associated viremia [9,10]. It has been reported that vaccination in horses causes an inflammatory response, characterized by an increase in acute-phase proteins that stimulate innate immune responses in the absence of clinical signs of inflammation [11]. Vaccines determine homeostatic changes as a response to inflammation, and are responsible for proinflammatory cytokine production, which determines a reduction in hepatic albumin synthesis in favor of the synthesis of acute-phase proteins such as serum amyloid A (SAA). Acute inflammatory reactions are indispensable for inducing the immune responses necessary for vaccinations to begin immunity [12,13].

Based the above, it is very important to consider possible homeostatic changes occurring after vaccination in horses undergoing competition, both for a temporary reduction in performance and for possible cardiac involvement secondary to the onset of inflammation.

Cardiac arrhythmias occur frequently in horses and are associated with many cardiac and noncardiac diseases. Most horses show arrhythmias following hypoxia, metabolic and electrolytic disorders, septicemia, endotoxemia, neurological diseases, or treatment with antiarrhythmic drugs [14]. The clinical significance of arrhythmias remains unclear and is strongly related to presence of other findings [14].

This study aimed to identify the possible occurrence of myocardial involvement following vaccination against Herpesvirus 1, justified by a probably systemic inflammatory response.

For this purpose, the clinical and electrocardiographic findings were recorded in heathy horses before and after vaccination. Moreover, assays of cardiac troponin (cTnI), and inflammatory biomarkers (serum amyloid A (SAA) and C-reactive protein (CRP)) were performed.

## 2. Materials and Methods

### 2.1. Ethical Statement and Animals

The study was performed during an official sanitary routine inspection by the official veterinarian of the locally competent district authority for an assessment of welfare and vaccine prophylaxis. Given that all procedures conducted in this study were noninvasive and routine operations that any good handler would conduct as part of daily checks, the work did not need approval by an ethics committee. However, all horse owners were informed of the aim of the study and clinical procedures, and signed informed consent was obtained. The owners were assured that their identities would be kept confidential. The protocol of this study was carried out according to the standards recommended by Italian and European rules on animal welfare.

Eighteen healthy horses with a mean body weight of 500 ± 25 kg and a mean age of 13 ± 13.5 years consisting of 7 females and 11 males (1 Paint Horse, 1 Quarter Horse, 1 Haflinger, 1 Appaloosa, 1 Purebred Oriental, 1 Purebred Arabian, and 12 half-breeds) were included. Before starting the study, all horses had been clinically inspected. Measurement of rectal temperature, observation of the mucous membranes, palpation of lymph nodes, and auscultation of the cardiac area and lung fields were performed. Routine hematology and biochemistry analyses including an electrolyte assay were also performed. All animals were vaccinated with an intramuscular inoculation (1 mL) of Pneumoequine^®^ (Merial, France). Vaccinated animals were checked by an official veterinarian throughout and after the vaccination procedure. All horses were evaluated 1 day before the vaccination (D0), and 7 days (D1) and 14 days afterwards (D2). At D0, D1, and D2, a blood sample was collected for evaluation of cTnI, SAA, and CRP, and an electrocardiographic examination was performed.

A scoring system to categorize the clinical findings was applied. Hyperthermia, altered color of the apparent mucous membranes, an increased volume of explorable lymph nodes, tachycardia, and/or tachypnea were considered as variables. Each variable was scored from 0 to 1, according to the absence or presence of the clinical sign considered (Table 1).

### 2.2. Electrocardiographic Examination

An electrocardiographic examination was recorded using a 3-channel electrocardiographic recording system (CARDIOLINE Delta plus, Italy) for all horses. Electrocardiograms (ECGs) were recorded at a paper speed of 25 mm/s and an amplitude of 10 mm/mV. Tracings were obtained at rest with the horse in the quadrupedal stance and positioned so that the body weight was distributed over all four limbs. Before recording, the subject was allowed to acclimatize for approximately 10 minutes, and then alligator electrodes were placed. The area was sprinkled with ethyl alcohol to ensure good electrical contact between the body surface and the electrodes themselves. Four electrodes were placed on the skin with crocodile clips in the following way:

(i) The electrodes for the forelimbs were placed at the level of the elbow: right, red electrode; left, yellow electrode.
(ii) The electrodes for the hind limbs were placed at the level of the skin fold of the patella: right, black electrode; left, green electrode.

After performing the ECGs, a graded approach was applied for their evaluation:

(a) Technical aspects: paper speed (25 mm/s), calibration signal (1 cm/mV), and filter systems (25–35 Hz to eliminate disturbances caused by strong muscle tremors);
(b) Qualitative evaluation of the traces and identification of artifacts produced by electrical interference, muscle tremors, or respiratory movements.
(c) Identification and qualitative evaluation of the isoelectric line.
(d) Assessment of the heart rate.
(e) Evaluation of heart rhythm and possible arrhythmias: regular or irregular.
(f) Identification of P-QRS-T complexes in all derivatives (I, II, III, avR, avL, avF).

For each patient, the morphology, duration, and amplitude of the P wave, PQ interval, QRS complex, ST segment, and T wave were assessed in DII at D0, D1 and D2. The obtained values were compared with those reported by Ayala et al. [15].

### 2.3. Blood Sampling Procedures

Blood samples were collected from each horse by jugular venipuncture using vacutainer tubes containing ethylenediamine tetra-acetic acid (EDTA) (Terumo Corporation, Tokyo, Japan) at D0, D1, and D2. Subsequently, the samples were processed at the laboratory of the University Veterinary Teaching Hospital of Messina. The tubes from each patient containing a coagulation activator were centrifuged at 3500 rpm for 10 min to separate the serum, and stored at −20 °C. Measurement of cardiac troponin I (cTnI), serum amyloid A (SSA), and C-reactive protein (CRP) was performed using an ELFA (Enzyme-Linked Fluorescent Assay, Minividas, Biomerieux, Noventa Padovana, Italy), an ELISA kit (Thermofisher, Waltham, MA, USA), and the CRP: CLIA method (Maglumi 800, Medical System, Brescia, Italy), respectively, in accordance with the manufacturers’ instructions.

### 2.4. Statistical Analysis

Statistical analysis was performed using the SPSS package for Windows (version 17.0, SPSS, Inc., Chicago, IL, USA). The means and standard deviations of the clinical score (CS), electrocardiographic measurements, cTnI, SAA, and CRP were calculated. For each variable, its normal distribution was assessed using the D’Agostino–Pearson test. One-way repeated measures ANOVA with a post hoc Tukey’s comparison test was used to detect statistical differences over time for each variable examined. Moreover, all variables were analyzed by comparing horses showing cardiac abnormalities with horses not presenting these. To determinate the relationship between the different variables, Spearman’s rank test was used. Significance was set at *p* < 0.05. Furthermore, each variable was reported with its descriptive statistical analysis.

## 3. Results

No adverse events related to the product, skin reactions, or general signs were observed after vaccine injection. No abnormalities were found in the hematological and biochemistry analyses.

### 3.1. Clinical Score (CS)

No changes in the clinical examination parameters (i.e., body temperature, nutritional status, behavioral changes, heart rate, respiratory rate, depression, and/or hyperthermia) were recorded. The mean values of CS were 2.4 ± 0.85 at D0, 2.3 ± 0.76 at D1, and 2.43 ± 1.03 at D2. Although five horses showed clinical signs of hyperthermia (*n* = 3), altered mucous membranes (*n* = 2), and lymph adenomegaly (*n* = 1), no significant difference was evident when comparing CS at D0, D1, and D2 (D0 vs. D1, *p* = 0.56; D0 vs. D2, *p* = 0.37; and D1 vs. D2 *p* = 0.93). The mean CS for horses showing clinical abnormalities was 1.8 ± 0.75. In these animals, cardiac abnormalities were also detected.

### 3.2. Electrocardiographic Findings

In terms of the electrocardiographic parameters, no horse had abnormalities in P-wave duration and amplitude, the QRS complex, and the related segments and/or intervals (Table 2). All variables related to the ECG readings were within the reference ranges for the species under investigation [6]. No significant differences were observed when comparing different time points in the study (*p* > 0.05). Six performance horses (*n* = 2 males, *n* = 4 females) presented with premature atrial complexes. Two horses (11%) had premature atrial complexes (PAC) at D1 that persisted at the next check at D2, while at D2, another four (22%) horses presented with the same cardiac abnormality.

An electrocardiographic diagnosis of PAC was performed by considering the presence of a P wave with a different conformation (due to its origin away from the SA node) from that of a sinus rhythm. The QRS complex was normal, as it is conducted through the ventricles via the normal pathway (although aberrant conduction is possible). The R-R interval preceding the PAC was short, whereas the R-R interval following the PAC was normal. This permitted us to differentiate PACs from premature complexes of ventricular origin, which are always followed by a compensatory pause [16].

### 3.3. Cardiac Troponin I (cTnI) Concentrations

The mean values of cTnI (ng/L) were 3.7 ± 2.21, 4 ± 2.6, and 7.2 ± 2.8 at D0, D1, and D2, respectively. No statistically significant differences were observed regarding cTnI values between D0 and D1 (*p* = 0.16), between D0 and D2 (*p* = 0.16), and between D1 and D2 (*p* = 0.91). Horses presenting premature atrial complexes showed significantly different values of cTnI at D1 vs. D0 (mean = 8.2 ± 2.7 and 2.5 ± 1.8, respectively; *p* = 0.045) and D2 vs. D0 (mean = 11.7 ± 7.8 and 2.5 ± 1.8, respectively; *p* = 0.006). Four horses with PACs had cTnI concentrations above the reference ranges reported in the literature (0.4–11 ng/mL) [16,17].

### 3.4. Serum Amyloid A (SSA) Concentrations

The mean SAA values (mg/mL) were 2.25 ± 0.2.37 at D0, 12.1 ± 7.6 at D1, and 14 ± 3.9 at D2. Significant differences related to SAA values were observed between D0 and D1 (*p* = 0.03), and between D0 and D1 (*p* = 0.03). Moreover, horses presenting with premature atrial complexes showed significant values of SAA (*p* = 0.027) at D2 (mean 15.6 ± 3.3) comparing with horses not presenting cardiac arrhythmias (12.3 ± 2.5).

### 3.5. C-Reactive Protein (CRP) Concentrations

The mean CRP values (mg/L) were 5.57 ± 1.6, 9.5 ± 1.5, and 6.5 ± 1.7 at D0, D1, and D2, respectively. No statistically significant differences in CRP values were observed between D0 and D1 (*p* = 0.63), between D0 and D2 (*p* = 0.87), and between D1 and D2 (*p* = 0.49). No statically significant differences were detected when comparing horses with and without PACs.

### 3.6. Correlations among the Variables Considered (CS, cTnI, SAA, and CRP)

A statistically significant correlation was observed between CRP and SAA at D2 (*p* = 0.006) and between SAA and cTnI (*p* = 0.05) (Table 3).

## 4. Discussion

This study aimed to demonstrate a correlation between electrocardiographic abnormalities and EHV-1 vaccination.

Vaccination is a fundamental element of preventive medicine, but it is not without risk. The vaccine used in this study (Pneumoequine, Merial, France) rarely causes side-effects; the package leaflet states that vaccination may exceptionally manifest a state of hypersensitivity. In this evaluation of the 18 horses, it was reported that two (11%) subjects had premature atrial complexes at D1 that persisted at the next check-up at D2, while at D2, another four (22%) subjects had the same cardiac anomaly. Horses who manifested premature atrial complexes were 4 years old (*n* = 1), 16 years old (*n* = 1), 12 years old (*n* = 2), and 23 years old (*n* = 2) (mean = 15 years).

The electrocardiographic examination offers the greatest diagnostic potential in the case of cardiac arrhythmias.

Arrhythmias can be recorded as primary electrical disorders or secondary to numerous factors, including structural cardiac disease, metabolic and endocrine disturbances, systemic inflammation, vascular disorders, automatism defects, and toxic/drugs [18].

In this study, the presence of a type of arrhythmia known as PACs was detected.

PACs begin with impulses generated in the atrial myocardium from a site other than the sinoatrial node (i.e., an ectopic site) and prematurely. PACs are a relatively common arrhythmia in horses [16]. They are also reported in healthy horses, and no clinical significance is attributed to a single isolated PAC at rest [14,19,20]. Horses with frequent PACs at rest may develop supraventricular tachycardia (four or more PACs in a row) or atrial fibrillation during maximal exercise [18]. Atrial enlargement, inflammation, electrolyte abnormalities, hypoxia, pyrexia, or sepsis may predispose a horse to PACs [21,22].

All horses presenting PACs presented normal hematological, biochemistry, and electrolyte values. Clinical signs such as hyperthermia, altered mucous membrane color, and lymph adenomegaly were reported in three horses. Although it was not possible to perform echocardiography to detect atrial enlargement in horses with PACs, the presence of clinical abnormalities associated with a cTnI increase and PACs in horses that, before the vaccination, did not present any clinical findings should suggest a possible effect of EHV vaccination in the onset of primary or secondary cardiac damage.

cTnI is a globular protein, located in the myofibrils, which regulates muscle contraction through the action of its three subunits: subunit T (TnT), which links the troponin and tropomyosin complexes; subunit I (TnI), which blocks muscle contraction in the absence of calcium; and subunit C (TnC), which binds calcium [23,24]. In recent years, cTnI has gained interest as a clinical marker of myocardial injury in several species. cTnI is known to leak from damaged myocardial cells and is a sensitive and specific marker for myocardial injury in humans and horses [25,26,27,28].

In humans, Cardiac Troponin I is primarily used to diagnose myocardial infarction but is also elevated in other conditions such as myocarditis and sepsis [29].

In human medicine, Sano et al. analyzed the incidence of electrocardiographic abnormalities during smallpox vaccination and demonstrated the presence of T-wave and S-T-segment abnormalities [30].

Another more recent study related to SARS-CoV-2 vaccination observed that in patients after administration of the Moderna vaccine, an abnormal heart rhythm and an increase in cTnI and CRP were found [31].

The data obtained suggested a possible direct relationship between the vaccination response and myocardial damage as measured by cTnI, which is considered to be an indicator of myocardial cell necrosis, which was also worsened by an increase in SAA in horses with PACs.

SAA is acknowledged as the most sensitive indicator of an acute phase response in horses. Its concentration increases as early as 6 h after stimulation by infection or tissue injury and, within 24–48 h, can be up to 1000 times the initial concentration. When the inflammation resolves, the SAA concentration falls within 12 h 4 [31,32].

In healthy horses, the physiological value of serum SAA concentration is less than 10 μg/mL. Values between 10 and 50 μg/mL indicate weak/moderate inflammation, values between 50 and 400 μg/mL indicate significant inflammation, and clinically important inflammation appears at values above 400 μg/mL. We observed a significant (*p* = 0.027) increase in SAA concentration at D2 in horses with PACs [13,33].

SAA is thought to be an excellent indicator of inflammation. Its increase in horses with PACs and a cTnI increase could be indicative of myocardial inflammation following to EHV-vaccination.

CRP is a protein produced by the liver and is one of the acute-phase proteins, a group of proteins synthesized during the inflammatory state. It belongs to the pentraxin family, which are pentahedral proteins consisting of five identical monomeric subunits associated with Ca^2+^ ions. CRP has the role of binding to the surface of microbes (particularly phosphorylcholine), acts as an attachment complex for complement proteins, and stimulates macrophage-mediated phagocytosis [34].

CRP in horses has a pentameric structure, as revealed by electron microscopy, and binds to PCh in a Ca^2+^-dependent manner. Equine CRP has 204 amino acid residues and a molecular weight of ~118 kDa. Equine CRP is composed of five identical nonglycosylated and noncovalently associated subunits with a molecular weight of ~23 kDa. Equine CRP shows immunochemical cross-reactivity with human CRP [35,36].

Although the mean values of CRP at D1 were above the reference ranges (7.5 mg/L) [37], no significant differences were observed for all horses at different timepoints, or between horses with and without PACs.

The data reported here are possibly related to the circulating levels of CRP. In adult horses, CRP increases by up 3 to 6 times the baseline values 24 h after inflammatory stimuli, then returns to normal values within 3 to 5 days afterwards [37].

## 5. Conclusions

The data obtained here identified the possible onset of myocardial involvement following vaccination against EHV-1, probably linked to a systemic inflammatory response, confirmed by an increase in cTnI and SAA in horses with PACs.

In fact, the results of this study showed that vaccination induced a prominent SAA and cTnI response in horses with the presence of premature atrial complexes.

Although it was not possible to correlate SAA concentrations with antibody titers, it is considered appropriate to carry these out on a larger group of horses in the future. Indeed, the vaccine stimulated the immune system and thus the expression of cytokines, which may lead to an increased production of acute-phase proteins. Thus, acute-phase protein concentrations should provide a means of assessing the response of the innate immune system to vaccination or disease.

Further studies, however, are needed to identify how vaccination may affect myocardial function, and which biomarkers may be considered useful for diagnosis.

## Figures and Tables

**Table 1:** Scoring system used during clinical evaluations of the horses included in the study.

Variables	0	1
Hyperthermia	Absent (37.5–38.5 °C)	Present (>38.5 °C)
Mucous membranes	Normal	Altered
Lymph nodes	Normal	Increased
Heart rate (HR)	<40 bpm	>40 bpm
Respiratory rate	<16 arm	>16 arm

arm = respiratory acts per minute, bpm = beats per minute.

**Table 2:** Duration and amplitude of P waves, the QRS complex, and their segments and/or intervals.

	P Wave (s)	P Wave (mV)	P-Q (s)	QRS (s)	R Wave (mV)	S-T (s)	T Wave (s)	T Wave (mV)
D0	0.07 ± 0.02	0.17 ± 0.06	0.19 ± 0.08	0.042 ± 0.01	0.68 ± 0.03	0.25 ± 0.06	0.06 ± 0.03	0.37 ± 0.23
D1	0.08 ± 0.02	0.17 ± 0.06	0.23 ± 0.08	0.046 ± 0.01	0.70 ± 0.03	0.29 ± 0.07	0.07 ± 0.02	0.33 ± 0.17
D2	0.08 ± 0.03	0.17 ± 0.05	0.23 ± 0.1	0.048 ± 0.01	0.72 ± 0.03	0.29 ± 0.04	0.07 ± 0.02	0.42 ± 0.26

**Table 3:** Statistical correlations (r) among CS, cTnI, SAA, and CRP at D0, D1, and D2.

	D0				D1				D2			
	CS	cTnI	SAA	CRP	CS	cTnI	SAA	CRP	CS	cTnI	SAA	CRP
CS	-	0.23	0.16	0.18	-	0.21	0.14	0.17	-	0.10	0.24	0.65
cTnI	0.23	-	0.10	0.03	0.21	-	−0.25	0.22	0.10	-	0.08	0.71 *
SAA	0.16	0.10	-	0.30	0.14	−0.25	-	0.30	0.24	0.08	-	0.12 *
CRP	0.18	0.03	0.30	-	0.17	0.22	0.30	-	0.65	0.71 *	0.12 *	-

* *p* < 0.05.

## Data Availability

Data may be found contacting the corresponding author (michela.pugliese@unime.it).
